# Scoping reviews: reinforcing and advancing the methodology and application

**DOI:** 10.1186/s13643-021-01821-3

**Published:** 2021-10-08

**Authors:** Micah D. J. Peters, Casey Marnie, Heather Colquhoun, Chantelle M. Garritty, Susanne Hempel, Tanya Horsley, Etienne V. Langlois, Erin Lillie, Kelly K. O’Brien, Ӧzge Tunçalp, Michael G. Wilson, Wasifa Zarin, Andrea C. Tricco

**Affiliations:** 1 University of South Australia, UniSA Clinical and Health Sciences, Rosemary Bryant AO Research Centre, Playford Building P4-27, City East Campus, North Terrace Adelaide, 5000 South Australia; 2 Adelaide Nursing School, Faculty of Health and Medical Sciences, The University of Adelaide, 101 Currie St, Adelaide, 5001 South Australia; 3 The Centre for Evidence-based Practice South Australia (CEPSA): a Joanna Briggs Institute Centre of Excellence, Faculty of Health and Medical Sciences, The University of Adelaide, 5006 Adelaide, South Australia; 4 Department of Occupational Science and Occupational Therapy, University of Toronto, Terrence Donnelly Health Sciences Complex, 3359 Mississauga Rd, Toronto, Ontario L5L 1C6 Canada; 5 Rehabilitation Sciences Institute (RSI), University of Toronto, St. George Campus, 160-500 University Avenue, Toronto, Ontario M5G 1V7 Canada; 6 Knowledge Synthesis Group, Ottawa Hospital Research Institute, 1053 Carling Avenue, Ottawa, Ontario K1Y 4E9 Canada; 7 Southern California Evidence Review Center, University of Southern California, Los Angeles, CA 90007 USA; 8 Royal College of Physicians and Surgeons of Canada, 774 Echo Drive, Ottawa, Ontario K1S 5N8 Canada; 9 Partnership for Maternal, Newborn and Child Health (PMNCH), World Health Organisation, Avenue Appia 20, 1211 Geneva, Switzerland; 10 Sunnybrook Research Institute, 2075 Bayview Ave, Toronto, Ontario M4N 3M5 Canada; 11 Department of Physical Therapy, University of Toronto, St. George Campus, 160-500 University Avenue, Toronto, Ontario M5G 1V7 Canada; 12 Institute of Health Policy, Management and Evaluation (IHPME), University of Toronto, St. George Campus, 155 College Street 4th Floor, Toronto, Ontario M5T 3M6 Canada; 13 UNDP/UNFPA/UNICEF/WHO/World Bank Special Programme of Research, Development and Research Training in Human Reproduction (HRP), Department of Sexual and Reproductive Health and Research, World Health Organisation, Avenue Appia 20, 1211 Geneva, Switzerland; 14 McMaster Health Forum, McMaster University, 1280 Main Street West, Hamilton, Ontario L8S 4L8 Canada; 15 Department of Health Evidence and Impact, McMaster University, 1280 Main Street West, Hamilton, Ontario L8S 4L8 Canada; 16 Centre for Health Economics and Policy Analysis, McMaster University, 1280 Main Street West, Hamilton, Ontario L8S 4L8 Canada; 17 Knowledge Translation Program, Li Ka Shing Knowledge Institute, St. Michael’s Hospital, Unity Health Toronto, 209 Victoria Street, East Building, Toronto, Ontario M5B 1T8 Canada; 18 Epidemiology Division and Institute for Health Policy, Management, and Evaluation, Dalla Lana School of Public Health, University of Toronto, 155 College St, Room 500, Toronto, Ontario M5T 3M7 Canada; 19 Queen’s Collaboration for Health Care Quality Joanna Briggs Institute Centre of Excellence, School of Nursing, Queen’s University, 99 University Ave, Kingston, Ontario K7L 3N6 Canada

**Keywords:** Scoping reviews, Evidence synthesis, Research methodology, Reporting guidelines, Methodological guidance

## Abstract

Scoping reviews are an increasingly common approach to evidence synthesis with a growing suite of methodological guidance and resources to assist review authors with their planning, conduct and reporting. The latest guidance for scoping reviews includes the JBI methodology and the Preferred Reporting Items for Systematic Reviews and Meta-Analyses—Extension for Scoping Reviews. This paper provides readers with a brief update regarding ongoing work to enhance and improve the conduct and reporting of scoping reviews as well as information regarding the future steps in scoping review methods development. The purpose of this paper is to provide readers with a concise source of information regarding the difference between scoping reviews and other review types, the reasons for undertaking scoping reviews, and an update on methodological guidance for the conduct and reporting of scoping reviews.

Despite available guidance, some publications use the term ‘scoping review’ without clear consideration of available reporting and methodological tools. Selection of the most appropriate review type for the stated research objectives or questions, standardised use of methodological approaches and terminology in scoping reviews, clarity and consistency of reporting and ensuring that the reporting and presentation of the results clearly addresses the review’s objective(s) and question(s) are critical components for improving the rigour of scoping reviews.

Rigourous, high-quality scoping reviews should clearly follow up to date methodological guidance and reporting criteria. Stakeholder engagement is one area where further work could occur to enhance integration of consultation with the results of evidence syntheses and to support effective knowledge translation. Scoping review methodology is evolving as a policy and decision-making tool. Ensuring the integrity of scoping reviews by adherence to up-to-date reporting standards is integral to supporting well-informed decision-making.

## Introduction

Given the readily increasing access to evidence and data, methods of identifying, charting and reporting on information must be driven by new, user-friendly approaches. Since 2005, when the first framework for scoping reviews was published, several more detailed approaches (both methodological guidance and a reporting guideline) have been developed. Scoping reviews are an increasingly common approach to evidence synthesis which is very popular amongst end users [1]. Indeed, one scoping review of scoping reviews found that 53% (262/494) of scoping reviews had government authorities and policymakers as their target end-user audience [2]. Scoping reviews can provide end users with important insights into the characteristics of a body of evidence, the ways, concepts or terms have been used, and how a topic has been reported upon. Scoping reviews can provide overviews of either broad or specific research and policy fields, underpin research and policy agendas, highlight knowledge gaps and identify areas for subsequent evidence syntheses [3].

Despite or even potentially because of the range of different approaches to conducting and reporting scoping reviews that have emerged since Arksey and O’Malley’s first framework in 2005, it appears that lack of consistency in use of terminology, conduct and reporting persist [2, 4]. There are many examples where manuscripts are titled ‘a scoping review’ without citing or appearing to follow any particular approach [5–9]. This is similar to how many reviews appear to misleadingly include ‘systematic’ in the title or purport to have adhered to the Preferred Reporting Items for Systematic Reviews and Meta-Analyses (PRISMA) statement without doing so. Despite the publication of the PRISMA Extension for Scoping Reviews (PRISMA-ScR) and other recent guidance [4, 10–14], many scoping reviews continue to be conducted and published without apparent (i.e. cited) consideration of these tools or only cursory reference to Arksey and O’Malley’s original framework. We can only speculate at this stage why many authors appear to be either unaware of or unwilling to adopt more recent methodological guidance and reporting items in their work. It could be that some authors are more familiar and comfortable with the older, less prescriptive framework and see no reason to change. It could be that more recent methodologies such as JBI’s guidance and the PRISMA-ScR appear more complicated and onerous to comply with and so may possibly be unfit for purpose from the perspective of some authors. In their 2005 publication, Arksey and O’Malley themselves called for scoping review (then scoping study) methodology to continue to be advanced and built upon by subsequent authors, so it is interesting to note a persistent resistance or lack of awareness from some authors. Whatever the reason or reasons, we contend that transparency and reproducibility are key markers of high-quality reporting of scoping reviews and that reporting a review’s conduct and results clearly and consistently in line with a recognised methodology or checklist is more likely than not to enhance rigour and utility. Scoping reviews should not be used as a synonym for an exploratory search or general review of the literature. Instead, it is critical that potential authors recognise the purpose and methodology of scoping reviews. In this editorial, we discuss the definition of scoping reviews, introduce contemporary methodological guidance and address the circumstances where scoping reviews may be conducted. Finally, we briefly consider where ongoing advances in the methodology are occurring.

### What is a scoping review and how is it different from other evidence syntheses?

A scoping review is a type of evidence synthesis that has the objective of identifying and mapping relevant evidence that meets pre-determined inclusion criteria regarding the topic, field, context, concept or issue under review. The review question guiding a scoping review is typically broader than that of a traditional systematic review. Scoping reviews may include multiple types of evidence (i.e. different research methodologies, primary research, reviews, non-empirical evidence). Because scoping reviews seek to develop a comprehensive overview of the evidence rather than a quantitative or qualitative synthesis of data, it is not usually necessary to undertake methodological appraisal/risk of bias assessment of the sources included in a scoping review. Scoping reviews systematically identify and chart relevant literature that meet predetermined inclusion criteria available on a given topic to address specified objective(s) and review question(s) in relation to key concepts, theories, data and evidence gaps. Scoping reviews are unlike ‘evidence maps’ which can be defined as the figural or graphical presentation of the results of a broad and systematic search to identify gaps in knowledge and/or future research needs often using a searchable database [15]. Evidence maps can be underpinned by a scoping review or be used to present the results of a scoping review. Scoping reviews are similar to but distinct from other well-known forms of evidence synthesis of which there are many [16]. Whilst this paper’s purpose is not to go into depth regarding the similarities and differences between scoping reviews and the diverse range of other evidence synthesis approaches, Munn and colleagues recently discussed the key differences between scoping reviews and other common review types [3]. Like integrative reviews and narrative literature reviews, scoping reviews can include both research (i.e. empirical) and non-research evidence (grey literature) such as policy documents and online media [17, 18]. Scoping reviews also address broader questions beyond the effectiveness of a given intervention typical of ‘traditional’ (i.e. Cochrane) systematic reviews or peoples’ experience of a particular phenomenon of interest (i.e. JBI systematic review of qualitative evidence). Scoping reviews typically identify, present and describe relevant characteristics of included sources of evidence rather than seeking to combine statistical or qualitative data from different sources to develop synthesised results.

Similar to systematic reviews, the conduct of scoping reviews should be based on well-defined methodological guidance and reporting standards that include an a priori protocol, eligibility criteria and comprehensive search strategy [11, 12]. Unlike systematic reviews, however, scoping reviews may be iterative and flexible and whilst any deviations from the protocol should be transparently reported, adjustments to the questions, inclusion/exclusion criteria and search may be made during the conduct of the review [4, 14]. Unlike systematic reviews where implications or recommendations for practice are a key feature, scoping reviews are not designed to underpin clinical practice decisions; hence, assessment of methodological quality or risk of bias of included studies (which is critical when reporting effect size estimates) is not a mandatory step and often does not occur [10, 12]. Rapid reviews are another popular review type, but as yet have no consistent, best practice methodology [19]. Rapid reviews can be understood to be streamlined forms of other review types (i.e. systematic, integrative and scoping reviews) [20].

### Guidance to improve the quality of reporting of scoping reviews

Since the first 2005 framework for scoping reviews (then termed ‘scoping studies’) [13], the popularity of this approach has grown, with numbers doubling between 2014 and 2017 [2]. The PRISMA-ScR is the most up-to-date and advanced approach for reporting scoping reviews which is largely based on the popular PRISMA statement and checklist, the JBI methodological guidance and other approaches for undertaking scoping reviews [11]. Experts in evidence synthesis including authors of earlier guidance for scoping reviews developed the PRISMA-ScR checklist and explanation using a robust and comprehensive approach. Enhancing transparency and uniformity of reporting scoping reviews using the PRISMA-ScR can help to improve the quality and value of a scoping review to readers and end users [21]. The PRISMA-ScR is not a methodological guideline for review conduct, but rather a complementary checklist to support comprehensive reporting of methods and findings that can be used alongside other methodological guidance [10, 12–14]. For this reason, authors who are more familiar with or prefer Arksey and O’Malley’s framework; Levac, Colquhoun and O’Brien’s extension of that framework or JBI’s methodological guidance could each select their preferred methodological approach and report in accordance with the PRISMA-ScR checklist.

### Reasons for conducting a scoping review

Whilst systematic reviews sit at the top of the evidence hierarchy, the types of research questions they address are not suitable for every application [3]. Many indications more appropriately require a scoping review. For example, to explore the extent and nature of a body of literature, the development of evidence maps and summaries; to inform future research and reviews and to identify evidence gaps [2]. Scoping reviews are particularly useful where evidence is extensive and widely dispersed (i.e. many different types of evidence), or emerging and not yet amenable to questions of effectiveness [22]. Because scoping reviews are agnostic in terms of the types of evidence they can draw upon, they can be used to bring together and report upon heterogeneous literature—including both empirical and non-empirical evidence—across disciplines within and beyond health [23–25].

When deciding between whether to conduct a systematic review or a scoping review, authors should have a strong understanding of their differences and be able to clearly identify their review’s precise research objective(s) and/or question(s). Munn and colleagues noted that a systematic review is likely the most suitable approach if reviewers intend to address questions regarding the feasibility, appropriateness, meaningfulness or effectiveness of a specified intervention [3]. There are also online resources for prospective authors [26]. A scoping review is probably best when research objectives or review questions involve exploring, identifying, mapping, reporting or discussing characteristics or concepts across a breadth of evidence sources.

Scoping reviews are increasingly used to respond to complex questions where comparing interventions may be neither relevant nor possible [27]. Often, cost, time, and resources are factors in decisions regarding review type. Whilst many scoping reviews can be quite large with numerous sources to screen and/or include, there is no expectation or possibility of statistical pooling, formal risk of bias rating, and quality of evidence assessment [28, 29]. Topics where scoping reviews are necessary abound—for example, government organisations are often interested in the availability and applicability of tools to support health interventions, such as shared decision aids for pregnancy care [30]. Scoping reviews can also be applied to better understand complex issues related to the health workforce, such as how shift work impacts employee performance across diverse occupational sectors, which involves a diversity of evidence types as well as attention to knowledge gaps [31]. Another example is where more conceptual knowledge is required, for example, identifying and mapping existing tools [32]. Here, it is important to understand that scoping reviews are not the same as ‘realist reviews’ which can also be used to examine how interventions or programmes work. Realist reviews are typically designed to ellucide the theories that underpin a programme, examine evidence to reveal if and how those theories are relevant and explain how the given programme works (or not) [33].

Increased demand for scoping reviews to underpin high-quality knowledge translation across many disciplines within and beyond healthcare in turn fuels the need for consistency, clarity and rigour in reporting; hence, following recognised reporting guidelines is a streamlined and effective way of introducing these elements [34]. Standardisation and clarity of reporting (such as by using a published methodology and a reporting checklist—the PRISMA-ScR) can facilitate better understanding and uptake of the results of scoping reviews by end users who are able to more clearly understand the differences between systematic reviews, scoping reviews and literature reviews and how their findings can be applied to research, practice and policy.

### Future directions in scoping reviews

The field of evidence synthesis is dynamic. Scoping review methodology continues to evolve to account for the changing needs and priorities of end users and the requirements of review authors for additional guidance regarding terminology, elements and steps of scoping reviews. Areas where ongoing research and development of scoping review guidance are occurring include inclusion of consultation with stakeholder groups such as end users and consumer representatives [35], clarity on when scoping reviews are the appropriate method over other synthesis approaches [3], approaches for mapping and presenting results in ways that clearly address the review’s research objective(s) and question(s) [29] and the assessment of the methodological quality of scoping reviews themselves [21, 36]. The JBI Scoping Review Methodology group is currently working on this research agenda.

Consulting with end users, experts, or stakeholders has been a suggested but optional component of scoping reviews since 2005. Many of the subsequent approaches contained some reference to this useful activity. Stakeholder engagement is however often lost to the term ‘review’ in scoping reviews. Stakeholder engagement is important across all knowledge synthesis approaches to ensure relevance, contextualisation and uptake of research findings. In fact, it underlines the concept of integrated knowledge translation [37, 38]. By including stakeholder consultation in the scoping review process, the utility and uptake of results may be enhanced making reviews more meaningful to end users. Stakeholder consultation can also support integrating knowledge translation efforts, facilitate identifying emerging priorities in the field not otherwise captured in the literature and may help build partnerships amongst stakeholder groups including consumers, researchers, funders and end users. Development in the field of evidence synthesis overall could be inspired by the incorporation of stakeholder consultation in scoping reviews and lead to better integration of consultation and engagement within projects utilising other synthesis methodologies. This highlights how further work could be conducted into establishing how and the extent to which scoping reviews have contributed to synthesising evidence and advancing scientific knowledge and understandings in a more general sense.

Currently, many methodological papers for scoping reviews are published in healthcare focussed journals and associated disciplines [6, 39–43]. Another area where further work could also occur is to gain greater understanding on how scoping reviews and scoping review methodology is being used across disciplines beyond healthcare including how authors, reviewers and editors understand, recommend or utilise existing guidance for undertaking and reporting scoping reviews.

## Conclusion

Whilst available guidance for the conduct and reporting of scoping review has evolved over recent years, opportunities remain to further enhance and progress the methodology, uptake and application. Despite existing guidance, some publications using the term ‘scoping review’ continue to be conducted without apparent consideration of available reporting and methodological tools. Because consistent and transparent reporting is widely recongised as important for supporting rigour, reproducibility and quality in research, we advocate for authors to use a stated scoping review methodology and to transparently report their conduct by using the PRISMA-ScR. Selection of the most appropriate review type for the stated research objectives or questions, standardising the use of methodological approaches and terminology in scoping reviews, clarity and consistency of reporting and ensuring that the reporting and presentation of the results clearly addresses the authors’ objective(s) and question(s) are also critical components for improving the rigour of scoping reviews. We contend that whilst the field of evidence synthesis and scoping reviews continues to evolve, use of the PRISMA-ScR is a valuable and practical tool for enhancing the quality of scoping reviews, particularly in combination with other methodological guidance [10, 12, 44]. Scoping review methodology is developing as a policy and decision-making tool, and so ensuring the integrity of these reviews by adhering to the most up-to-date reporting standards is integral to supporting well informed decision-making. As scoping review methodology continues to evolve alongside understandings regarding why authors do or do not use particular methodologies, we hope that future incarnations of scoping review methodology continues to provide useful, high-quality evidence to end users.

## Data Availability

All data and materials are available upon request.
